# 4183 Micro-consults: An effective tool for meeting statistical support needs in an academic medical research center

**DOI:** 10.1017/cts.2020.181

**Published:** 2020-07-29

**Authors:** Sandra Taylor, Susan L. Stewart

**Affiliations:** 1 University of California, Davis

## Abstract

OBJECTIVES/GOALS: Access to biostatistics expertise is essential for a successful clinical and translational research program. However, demand for statistical support at academic research centers can strain the capacity of biostatistics units. Our objective was to efficiently increase access to statistical expertise. METHODS/STUDY POPULATION: In cooperation with the Cancer Center Biostatistics Shared Resource, we replaced an informal 1-hour drop-in consultation program with structured office hours to provide statistical support to clinical and translational researchers at the University of California, Davis Medical Center. We doubled office hours to 2 hours per week and established six 20-minute appointments. Two Ph.D. level statisticians staff office hours. Researchers schedule appointments through Acuity Scheduling, a free on-line resource. Availability of the service is advertised monthly by sending an informational flyer to various university listservs. RESULTS/ANTICIPATED RESULTS: Prior to implementing the program in 2014, we averaged 91 office hour consults per year. Subsequently, consultations jumped to 171 in 2014 and have averaged 150 per year since then. Office hours attract students, residents, staff and faculty from a wide range of disciplines including the Schools of Medicine, Nursing, Veterinary Medicine and basic science departments. Project types span the clinical and translational spectrum covering lab, animal, clinical and population-level studies. Most consults related to data analysis and interpretation (57%) followed by sample size calculations/study design (29%) and response to reviewers (4%), with general statistical advice as the remainder. DISCUSSION/SIGNIFICANCE OF IMPACT: With 6 micro-consults per week, we can meet with many investigators and triage their statistical support needs. This program has proved very popular and was highly rated in a recent user survey, with several investigators noting that the consults facilitated successful publications and proposals.

